# Supplementary material for: Controllable ion transport by surface-charged graphene oxide membrane
Source: Nat Commun. 2019 Mar 19;10:1253. doi: 10.1038/s41467-019-09286-8 (PMC6424959; doi:10.1038/s41467-019-09286-8)
Supplement: Supplementary file 1 — Supplementary Information [file 41467_2019_9286_MOESM1_ESM.pdf]

Supplementary Information

for

Controllable ion transport by surface-charged graphene oxide membrane

Zhang et al.

This file includes:

Supplementary Figures. 1 to 25

Supplementary Tables 1 to 4

Supplementary References 1 to 35

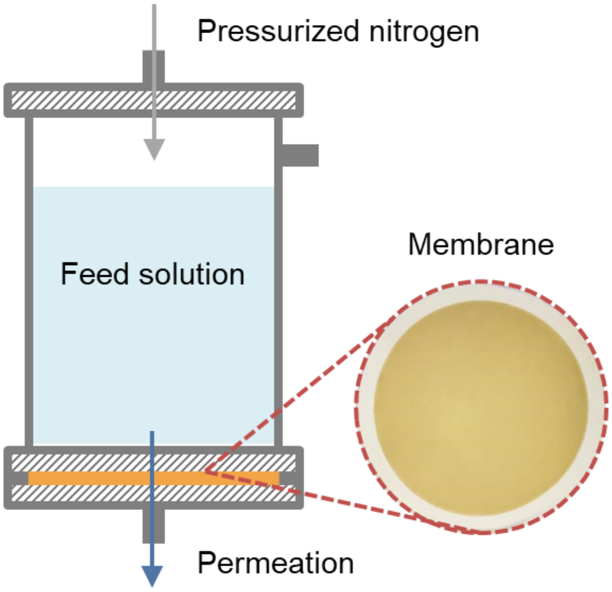

Supplementary Figure 1 Schematic of self-designed filtration device.

The membrane preparation system is basically shared with the performance evaluation using this self-designed filtration set up showing in Supplementary Figure 1. Briefly, the membrane (marked as orange) is mounted into the device and sealed by rubber sealing ring (marked as gray) to avoid leakage. The feed solution (prepared solution or tested solution) in the upper container is controllably drove by pressured nitrogen and permeates through the membrane. The permeation is collected at the bottom and analyzed to determine the separation performance of the membranes.

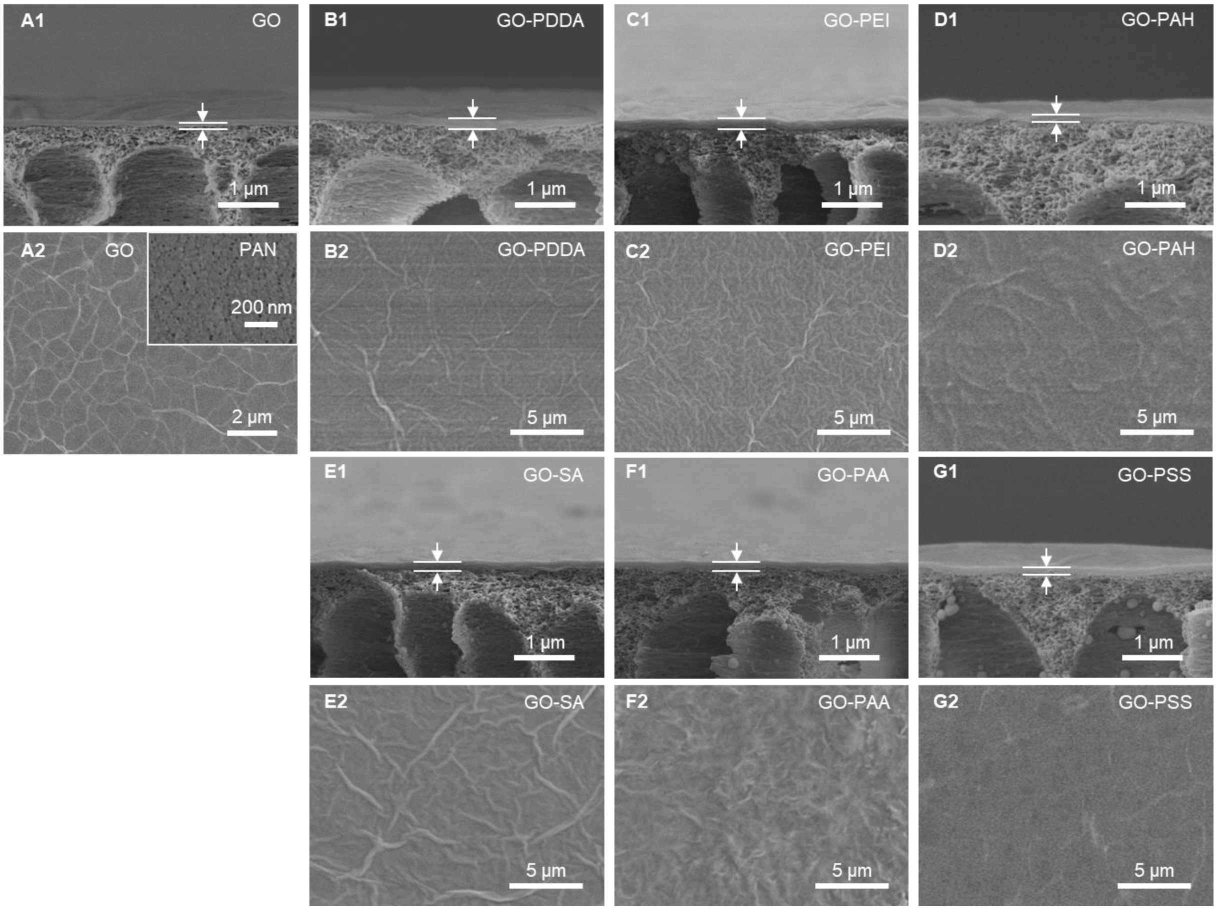

Supplementary Figure 2 SEM surface morphology and cross-sectional view of **(A)** pristine GO membrane (GO deposition amount of 0.5 mg; Insert: SEM image of PAN substrate surface; membrane diameter of 4.7 cm with effective area of ~17.35 cm^2) and **(B-G)** surface-charged GO membranes (GO deposition amount of 5 mg with 0.1 wt% PDDA, PEI, PAH, SA, PAA, PSS polyelectrolytes surface coating respectively; membrane diameter of 4.7 cm with effective area of ~17.35 cm^2).

As observed in Supplementary Figure 2, the GO nanosheets well assemble and fully cover the porous PAN substrate forming a defect-free laminar membrane. The membrane surface reveals apparent surficial ripples and fluctuations, which arise from the formation of convoluted and folded structure of nanosheets during assembly[1]. The morphological changes of surface-charged GO membranes as compared to pristine GO membrane are attributed to the filling of polyelectrolyte in the low-lying of the GO wrinkles. Some small bulges covering the membranes surface are observed due to the chemical conformation and the aggregation of the top

polyelectrolytes layer, indicating the successful coating of polyelectrolytes on surface of GO membranes[2].

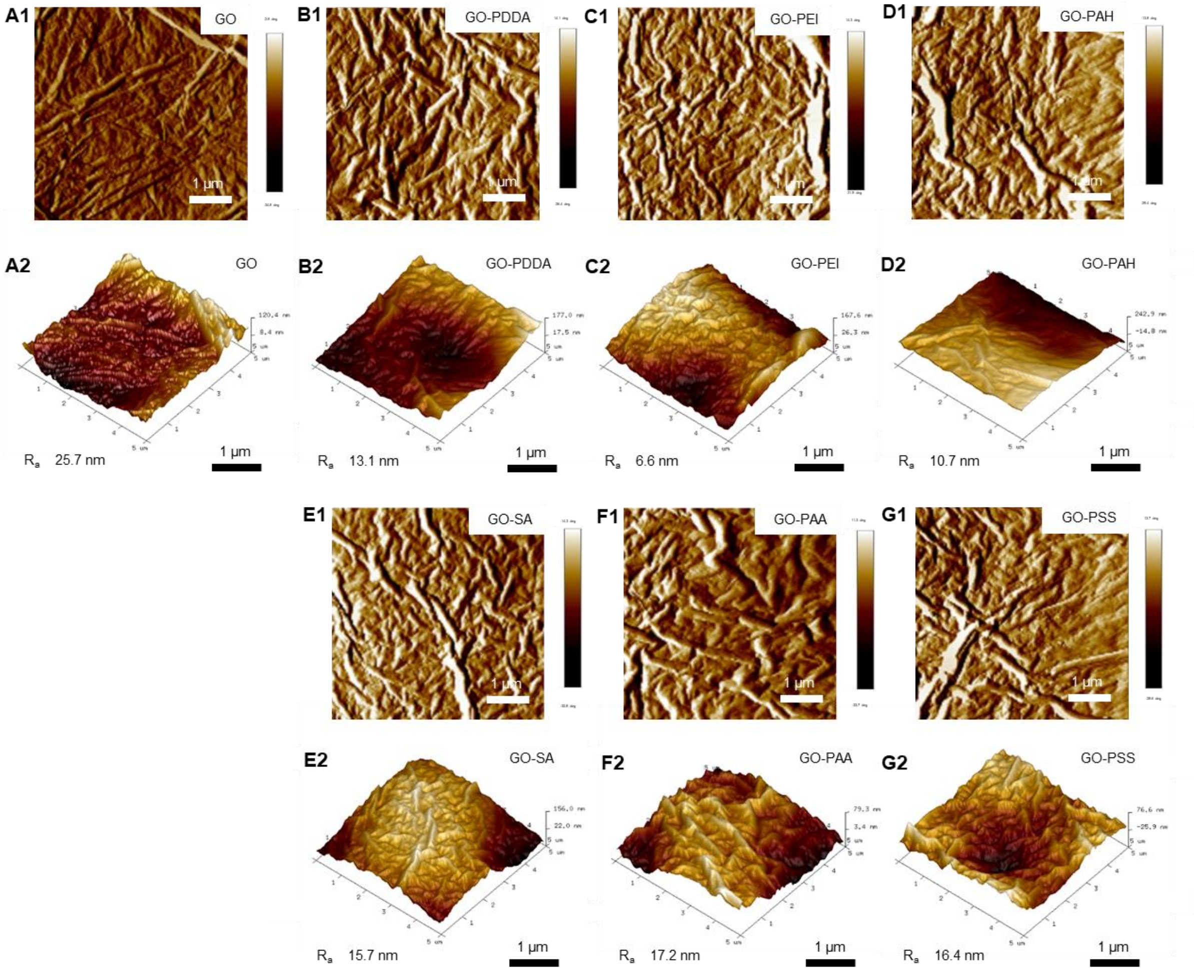

Supplementary Figure 3 AFM phase and height images of **(A)** pristine GO and **(B-G)** surface-charged GO membranes.

Supplementary Figure 3 gives AFM phase and height images of pristine GO and surface-charged GO membranes. We can see from the three-dimensional height images that the GO membranes surface exhibit crumpled and wrinkled structure, which is consistent with the morphologies of membranes in SEM images. The roughness of membranes top surface decrease after coating polyelectrolytes. In addition, the AFM phase images show apparent two phases on the surface of these membranes, except for the case of pristine GO membrane, which has only one phase. A dark-colored area refers to a rigid GO phase, and a light-colored area pertains to a

soft polymer phase[3]. From a comparison of the phase and height images, we can identify that the white strips refer to the polyelectrolytes, confirming the uniform attachment of polyelectrolytes on GO membrane surface.

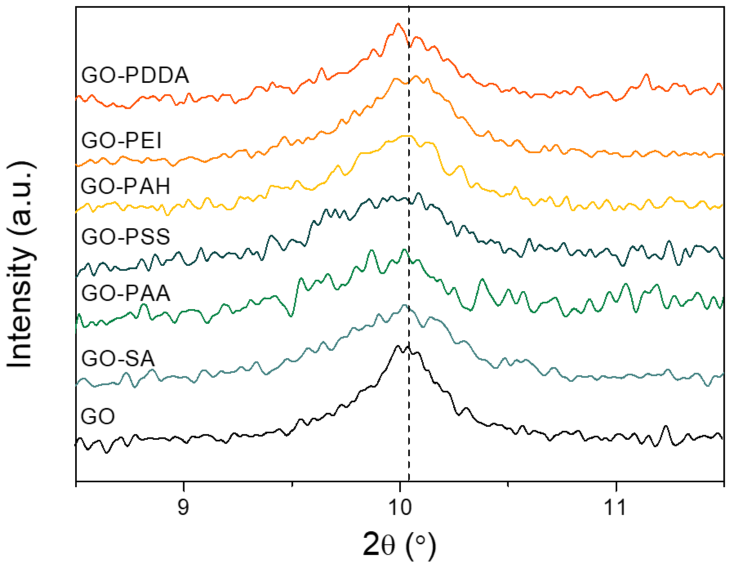

Supplementary Figure 4 XRD patterns of pristine GO and surface-charged GO membranes.

The XRD patterns of pristine GO and surface-charged GO membranes in Supplementary Figure 4 show almost same 2θ values of ~10.1° corresponding to typical d-spacing of ~8.7 Å, which demonstrate that the ordered laminar structure of GO layer in surface-charged GO membranes remains unchanged[4].

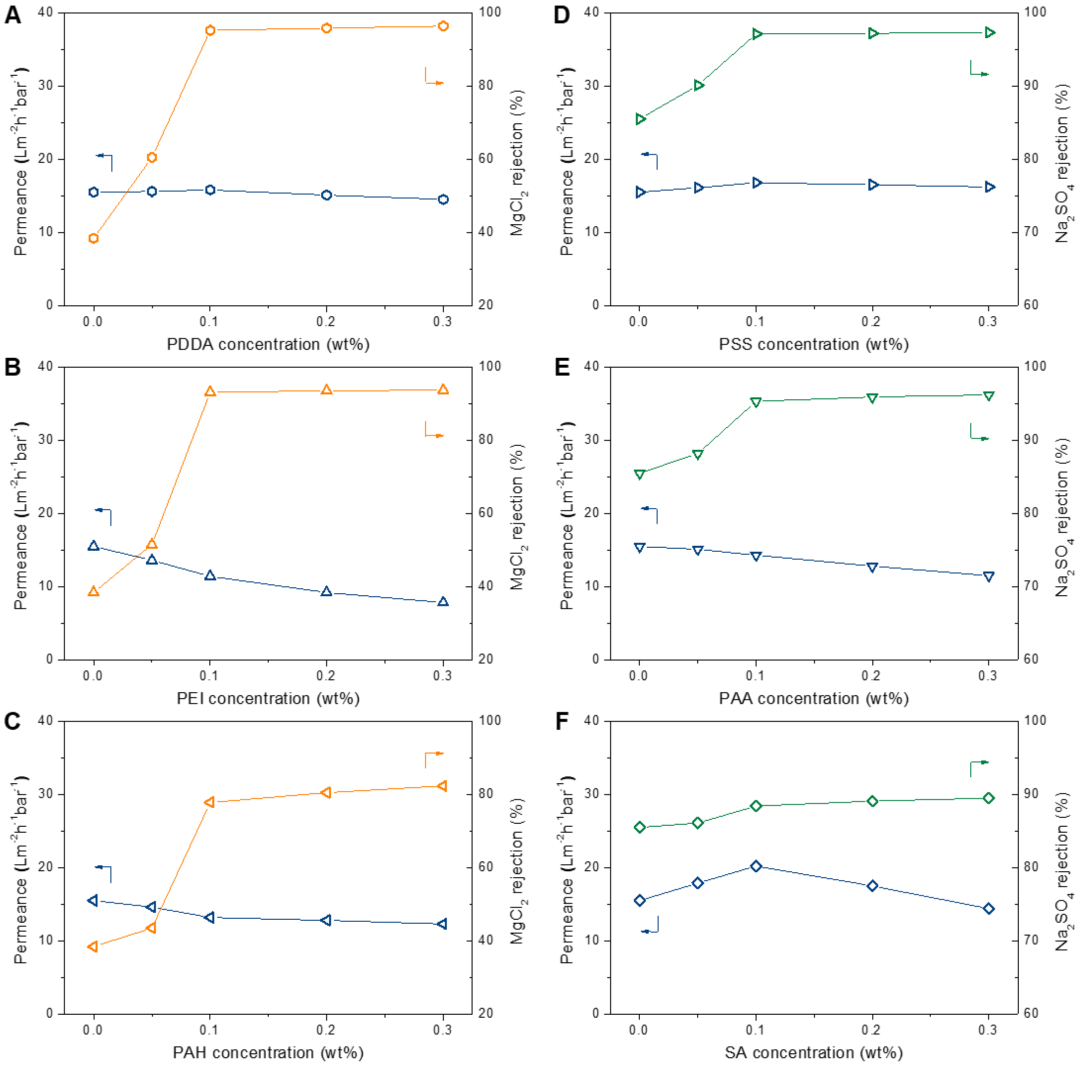

Supplementary Figure 5 Water permeance and salt rejections of surface-charged GO membranes with different polyelectrolyte coating concentrations.

The water permeance and salt rejections of surface-charged GO membranes with different polyelectrolyte coating concentrations were investigated to find optimized preparation conditions and validate the formation of polyelectrolyte layers. Supplementary Figure 5 shows the rise of rejections turns to stable, which demonstrates that the polyelectrolyte layer forms from loose to dense covering of membrane surface, and the provided surface charge trends to the instinct charge of polyelectrolyte itself. The changes in water permeance of the membranes dependent on the restricting relations between improved hydrophilic property and enhanced water transport resistance. The optimized polyelectrolyte coating concentration is 0.1 wt%.

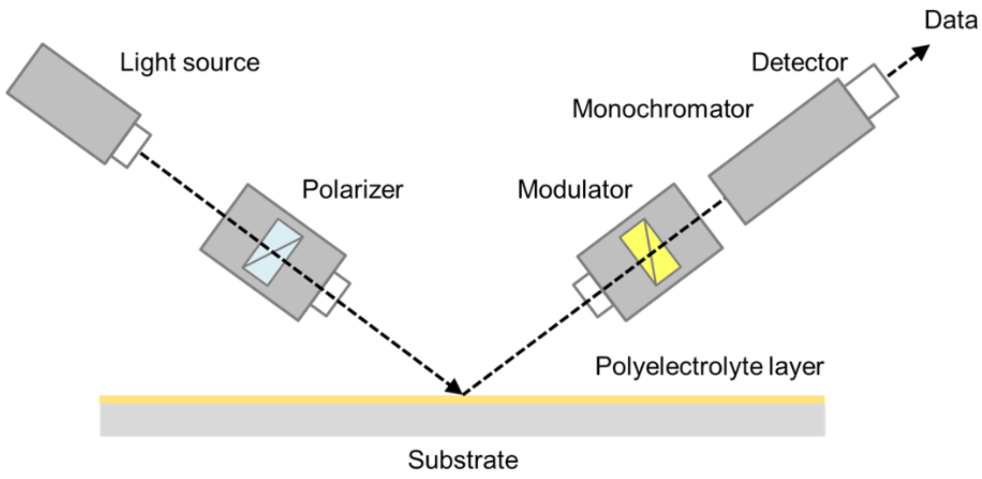

Supplementary Figure 6 Schematic of ellipsometer for polyelectrolyte layer thickness measurement.

Supplementary Table 1 Thickness of polyelectrolyte layers measured by ellipsometry.

Sample	PDDA	PEI	PAH	SA	PAA	PSS
Thickness (nm)	25±2	38±5	29±3	36±5	32±4	27±2

Ellipsometry is an optical technique for investigating sample thickness. As illustrated in Supplementary Figure 6, electromagnetic radiation is emitted by a light source and linearly polarized by a polarizer, and then falls onto the sample. After reflection the radiation passes a modulator and a monochromator, and finally falls into the detector. The sample thickness can be calculated by further performing a model analysis. In order to obtain the physical thickness of polyelectrolyte layers, we prepared samples by coating polyelectrolytes on the surface of Si wafers using 0.1 wt% polyelectrolyte solutions. The results are summarized in Supplementary Table 1. Error bars represent standard deviations for 3 measurements.

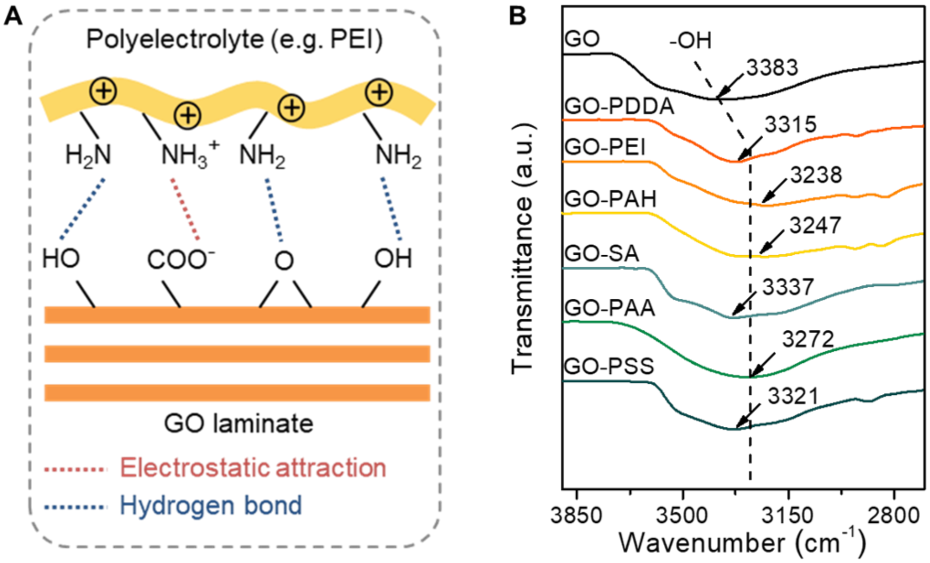

Supplementary Figure 7 (A) Schematic of probing interactions between polyelectrolyte layer and GO laminate. **(B)** Peak shifting in FTIR spectra of GO and surface-charged GO membranes.

The chemical binding between polyelectrolyte layer and GO laminate could be formed through abundant interaction sites, which includes hydrogen bonds among functional groups and electrostatic attraction between polycations and negatively charged GO, as shown in Supplementary Figure 7A (taking GO-PEI membrane as an example). The redshifts of –OH peaks in FTIR spectra in Supplementary Figure 7B of surface-charged GO membranes confirm the formation of hydrogen bonds[5].

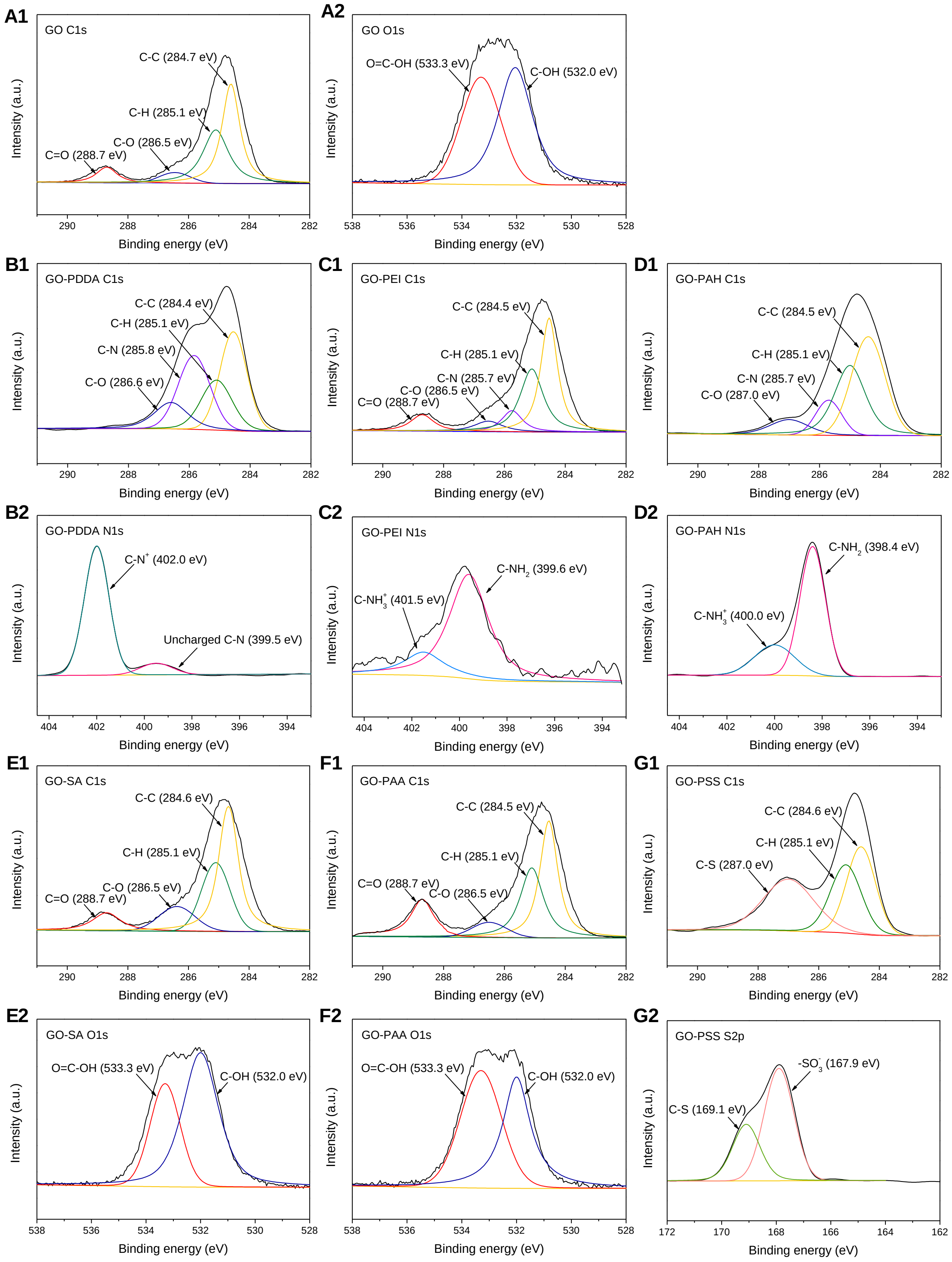

Supplementary Figure 8 XPS C 1s and O 1s (N 1s or S 2p) spectra of **(A)** pristine GO and **(B)** surface-charged GO membranes.

The surface composition of the membranes and their functional speciation can be clarified by XPS analysis. Supplementary Figure 8 gives the XPS C 1s, and O 1s (N 1s or S 2p) spectra of pristine GO and surface-charged GO membranes. The contributions of pristine GO membrane in C 1s spectra at ~284.7 eV, ~286.5 eV and ~288.7 eV arise from the C-C (sp^3 bonded carbons), alkoxyl and epoxyl C-O, carbonyl and carboxy C=O moieties, respectively. The O 1s spectra could be fitted with two contributions arising from O=C-OH at 533.3 eV and C-OH at 532.0 eV[5].

The C 1s spectra of GO-PDDA, GO-PEI and GO-PAH membranes clearly show new peak at ~285.7 eV of C-N moieties though partly overlapped with the C-O contributions, confirming the presence of amino groups on the membranes. In addition, the peak intensity of C-N moieties in the C 1s spectrum of GO-PDDA is apparently higher than the two others. The N 1s spectra of GO-PDDA show contributions arising from the charged and uncharged quaternary amine moieties; and GO-PEI and GO-PAH membranes display characteristic peaks corresponding to charged and uncharged primary amine moieties[6].

In the C 1s spectra of GO-SA and GO-PAA membranes, strikingly, compared to pristine GO membrane, GO-SA and GO-PAA membranes are characterized by stronger contributions at ~286.5 eV and ~288.7 eV, respectively. These observations are indicative of the larger amount of -OH and -COOH groups on GO-SA and GO-PAA membranes, respectively. The O 1s spectra of them further verified the varied contents of carboxyl and hydroxyl groups on the membranes. The O 1s spectra can be fitted with two contributions arising from O=C-OH (~533.3 eV) and C-OH (~532.0 eV), respectively. The intensity variation of the two peaks clearly evidenced the effective introduction of additional hydroxyl and carboxyl groups on GO-SA and GO-PAA membranes, respectively. The new peak corresponding to C-S linkage shows at ~387.0 eV in the C 1s spectrum of GO-PSS membrane, while the characteristic peaks at ~169.1 eV and ~167.9 eV can be assigned to S-C and $-SO_3$ groups, indicating the presence of sulfonic acid groups[7].

Notice that numerous hydrogen bonds formed between different functional groups on GO and polyelectrolytes, leading to peaks shifting. For example, the C 1s component peaks shift from 284.7 eV (C-C) and 286.5 eV (C-O) in pristine GO membrane to 284.4 eV and 287.0 eV in surface-charged GO membranes, respectively. These results suggest the formation of hydrogen bonds among functional groups of GO and polyelectrolytes[5].

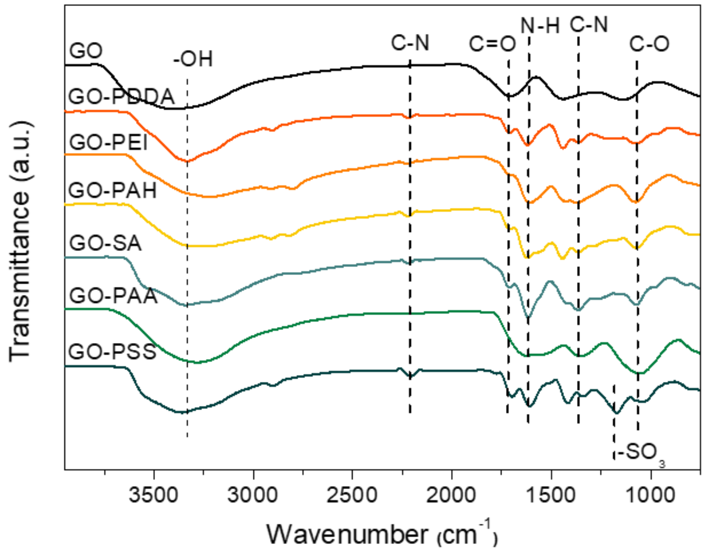

Supplementary Figure 9 FTIR spectra of pristine GO and surface-charged GO membranes.

To verify the formation of surface polyelectrolyte layers, FTIR spectra of pristine GO and surface-charged GO membranes are compared in Supplementary Figure 9. The pristine GO membrane has characteristic peaks of -OH stretching at ~3400 cm^{-1}, C=O stretching at ~1700 cm^{-1}, C-O stretching at ~1060 cm^{-1}, which show the typical chemical structure of GO. After coating with polyelectrolytes, new peaks appear on the FTIR spectra of GO-PDDA, GO-PEI and GO-PAH membranes at 2223 cm^{-1}, 1367 cm^{-1} and 1617 cm^{-1}, which can be ascribed to C-N bond in secondary amine and $–NH_2$ group of primary amines, respectively. The intensity of broad peak at 3300 cm^{-1} on the FTIR spectrum of GO-SA membrane increases due to the abundant hydroxyl groups after coating with SA. The enhanced peaks at 1720 cm^{-1} on the FTIR spectrum of GO-PAA membrane belong to C=O, indicating a large amount of carboxyl groups on the membrane surface. The new peak at 1178 cm^{-1} represented $–SO_3$ can be observed on the FTIR spectrum of GO-PSS membrane.

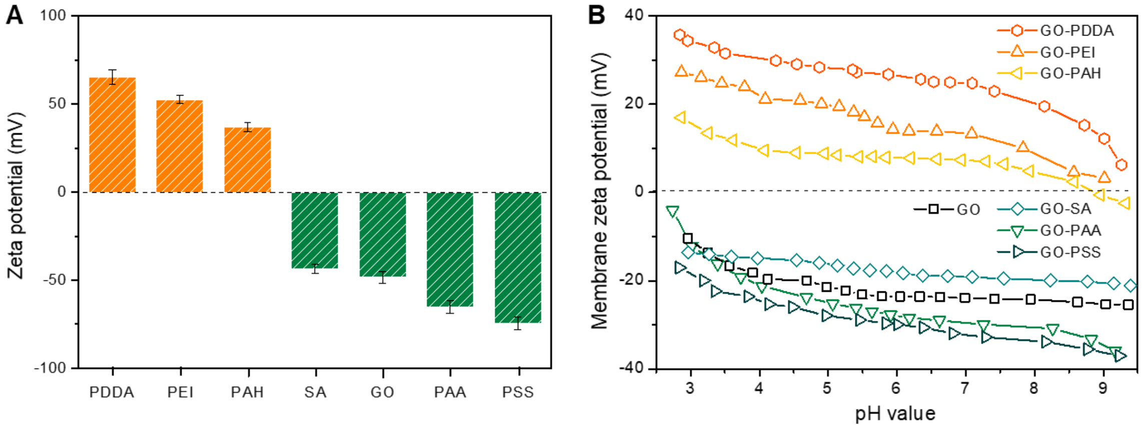

Supplementary Figure 10 Zeta potentials of **(A)** GO and polyelectrolyte aqueous solutions (0.1 mg/mL, pH 7), and **(B)** GO, GO-PAA, GO-PEI, GO-PAH, GO-PSS, GO-PAA and GO-SA membranes within the pH range from 2.5 to 9.5. Error bars represent standard deviations for 3 measurements.

The zeta potentials of GO and polyelectrolyte aqueous solutions at neutral pH are shown in Supplementary Figure 10A. It reveals that PDDA, PEI and PAH are positively charged due to the protonation of amine groups, while SA, GO, PAA and PSS are negatively charged due to deprotonating of carboxyl and hydroxyl groups. The absolute value difference of zeta potentials of these materials is determined by the intensity and amount of ionizable functional groups. Supplementary Figure 10B illustrates the surface zeta potential curves of the seven membranes at pH values ranging from 2.5 to 9.5. It is found that pristine GO membrane is able to maintain negatively charged over the whole pH range with surface charge density varying. For polycations coating GO membranes (i.e. GO-PDDA, GO-PEI and GO-PAH), because additional amine groups are introduced on the surface, the membrane is positively charged with high charge density. On the contrary, compare to pristine GO membrane, the zeta potential curve of polyanions coating GO membranes (i.e. GO-PSS and GO-PAA) move down, implying that the polyanions can make the membrane surface more negatively charged. However, a more flattened curve is found for the GO-SA membrane showing a less surface charge, because of the weak deprotonation ability of surface hydroxyl groups.

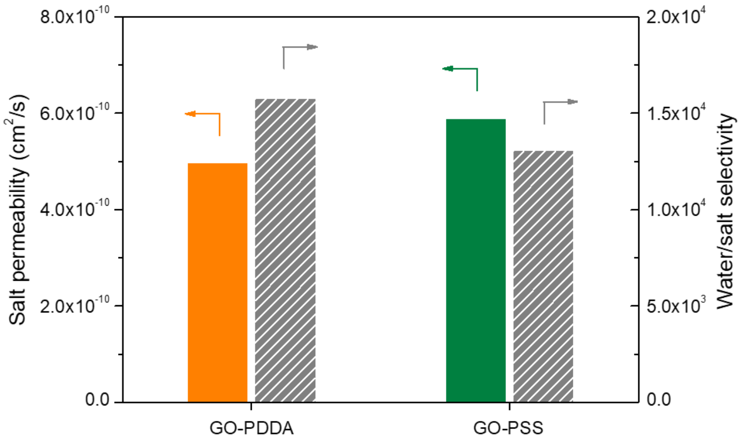

Supplementary Figure 11 Salts permeability and water/salt selectivity of surface-charged GO membranes for NaCl.

As shown in Supplementary Figure 11, the positively charged GO-PDDA and negatively charged GO-PSS membranes have similar transport behaviors for NaCl, which indicates the balance of electrostatic repulsion against monovalent co-ions and electrostatic attraction towards counter-ions in both positively and negatively charged GO membranes. Although there is a balanced electrostatic interaction with co-ions and counter-ions in the case of $MgSO_4$ with Z^+/Z^- =1, the difference in hydrated ionic radius of Mg^{2+} (0.428 nm) and SO_4^{2-} (0.397 nm) would also affect the permeation of $MgSO_4$ through the surface-charged GO membranes. The positively charged GO membrane tends to repel Mg^{2+} and attract SO_4^{2+}, in which the smaller SO_4^{2+} is easier to pass through the membrane thus leading to a relatively higher salt permeability. Conversely, the larger Mg^{2+} yields greater steric hindrance to transport through the negatively charged GO membrane, resulting in a relatively lower salt permeability.

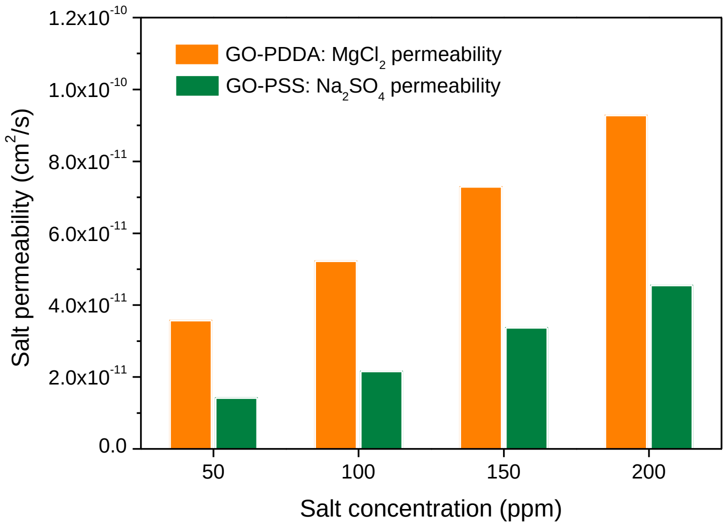

Supplementary Figure 12 $MgCl_2$ permeability of positively charged GO-PDDA membrane and Na_2SO_4 permeability of negatively charged GO-PSS membrane under salt concentrations ranging from 50-200 ppm.

$MgCl_2$ (or Na_2SO_4) permeability of positively (or negatively) charged GO membrane increases with salt concentration in feed solution ranging from 50-200 ppm. It is because the higher charge density around membrane surface attracts more counter-ions into the membrane surface and pores, which would neutralize partial membrane surface charges thus weakening the charge repulsion to the co-ions. This greater charge screening effect at high salt concentration lessens the exclusion effect, finally causing the increase of salt permeability[8,9].

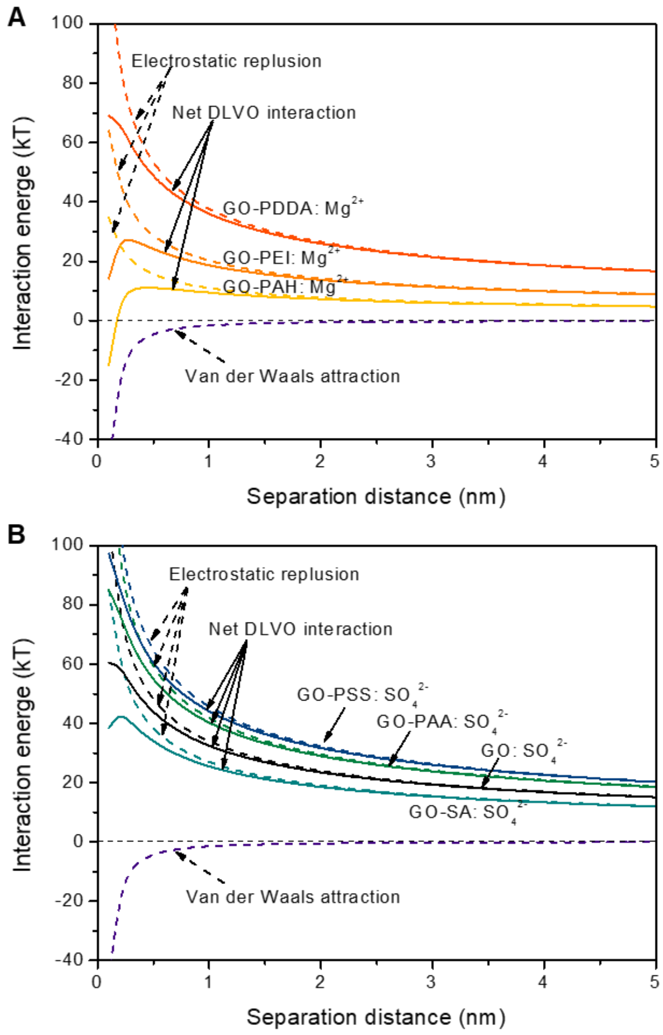

Supplementary Figure 13 Surface element integration model predictions of Derjaguin-Landau-Verwey-Overbeek (DLVO) interaction energies between ions and **(A)** positively charged GO and **(B)** negatively charged GO membranes surface by adding Van der Waals attraction and electrostatic repulsion.

The results of DLVO interactions between charged ions and surface-charged GO membranes indicate that the Van der Waals attraction is fixed, while the electrostatic repulsion is positively correlated with membrane surface zeta potential. The net DLVO interaction energy intrinsically embodies the repulsive capability to exclude target ions, which can well explain that the surface-charged GO membranes can create favorable electrostatic repulsion against co-ions so that realizing controllable ions transport.

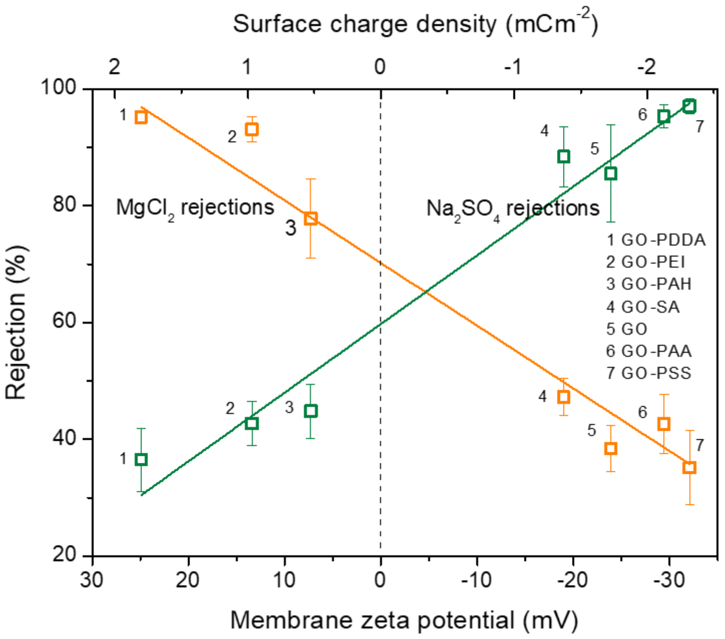

Supplementary Figure 14 $MgCl_2$ and Na_2SO_4 rejections of positively and negatively charged GO membranes with various membrane zeta potential (or membrane surface density). Error bars represent standard deviations for 3 measurements.

As observed in Supplementary Figure 14, the order of membrane salt rejections follows the sequence of membrane surface charges from highly positive to highly negative: rejection for AB_2-type salt (i.e., $MgCl_2$) from highest to lowest, while rejection for A_2B-type salt (i.e., Na_2SO_4) from lowest to highest. The two distinct trends highlight a key finding that highly positively charged GO membranes strongly reject salts containing divalent cations such as Mg^{2+}, while highly negatively charged GO membranes remarkably repel salts containing divalent anions such as SO_4^{2-}, which is well-consistent with the conclusions achieved in ions transport measurements. The high salts rejection enables the surface-charged GO membranes to be promising candidates for nanofiltration applications.

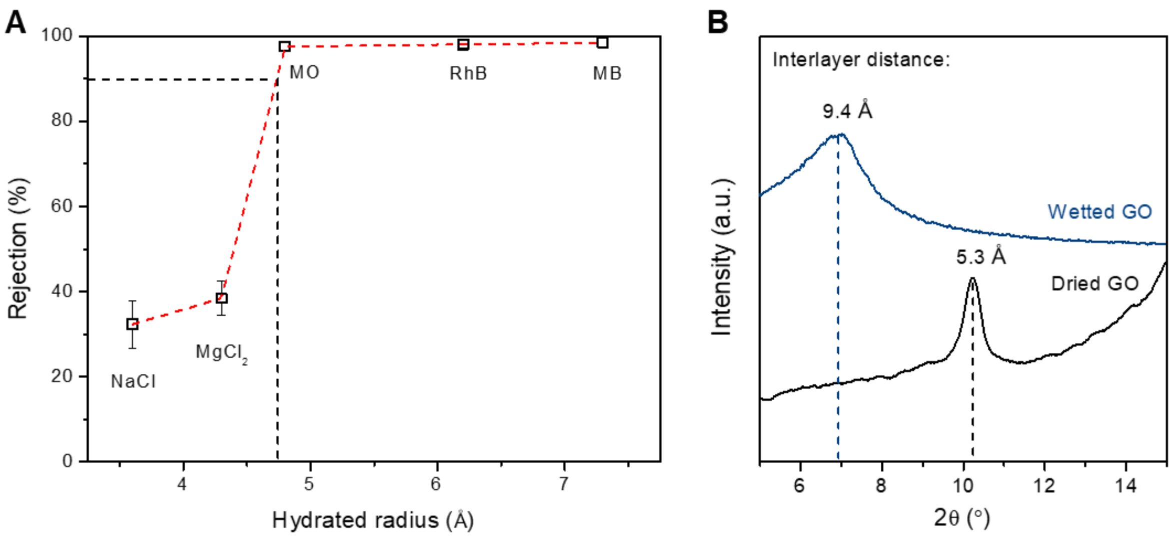

Supplementary Figure 15 (A) Experiments for salts (NaCl and $MgCl_2$) and dye molecules (methyl orange, MO; Rhodamine B, RhB; methylene blue, MB) retention of pristine GO membrane (GO deposition amount of 0.5 mg) as a function of their hydrated radius. The dotted lines mark the membrane molecular weight cut-off (MWCO) of ~4.7 Å. **(B)** XRD patterns for pristine GO membranes in dried and wetted states. The interlayer distance is obtained by subtracting thickness of pristine grapheme (3.4 Å) from d-spacing (calculated from 2θ value based on Bragg equation). Error bars represent standard deviations for 3 measurements.

We performed the filtration of several salts and dye molecules with different hydrated radius. The pristine GO membrane can well block dye molecules while insufficient to retain monovalent and divalent ions, showing a sharp sieving cut-off of hydrated radii ~4.7 Å. The XRD patterns compare the dried and wetted GO membranes with 2θ value switched from 10.1^{o} to 6.9^{o}, corresponding to the d-spacing of 8.7 Å and 12.8 Å, respectively. Subtracting the thickness of pristine grapheme (3.4 Å) from d-spacing, the interlayer distances for ionic and molecular transport are 5.3 Å (of dried GO membrane) and 9.4 Å (of wetted GO membrane). The enlarged interlayer distance of wetted GO membrane is due to the water intercalation, which is well-agreed with the sieving cut-off from the experimental result[10].

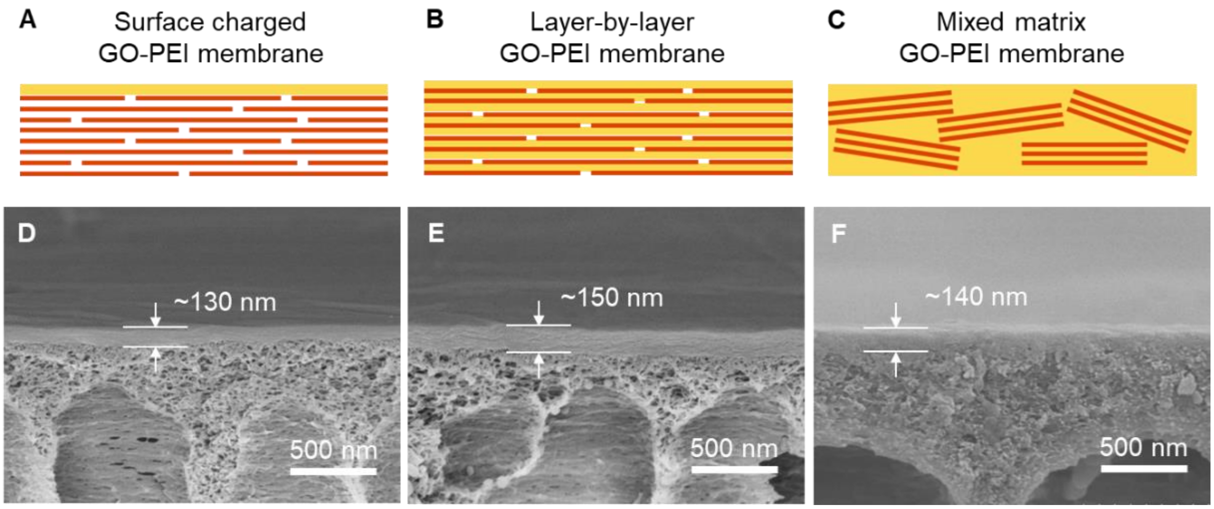

Supplementary Figure 16 Typical schematics and SEM cross sectional images of GO-PEI membranes with configuration of **(A)** surface-charged GO-PEI membrane (GO deposition amount of 0.5 mg with 0.1 wt% PEI solution surface coating for 30 min), **(B)** layer-by-layer GO-PEI membrane (alternately deposition of 10 mL GO suspension containing 0.025 mg GO and 10 mL 0.1 wt% PEI solution for 20 times via vacuum filtration) and **(C)** mixed matrix GO-PEI membrane (spin coating using 20 mL mixed preparing solution containing 0.5 mg GO and 0.5 g PEI).

As compared in Supplementary Figure 16, the three kinds of GO-PEI membranes with different configurations have similar membrane thickness. The surface-charged GO-PEI membrane shows integrated polyelectrolyte layer and GO laminate. Layer-by-layer GO-PEI membrane possesses a thicker and compacter laminar structure owning to the strong and abundant electrostatic attractions of positively charged PEI layer and negatively charged GO layer. In addition, the mixed matrix GO-PEI membrane has entirely different configuration with polymeric materials as mainbody, and shows serious pore penetration of small molecular weight PEI polyelectrolyte blocking the pores of PAN substrate.

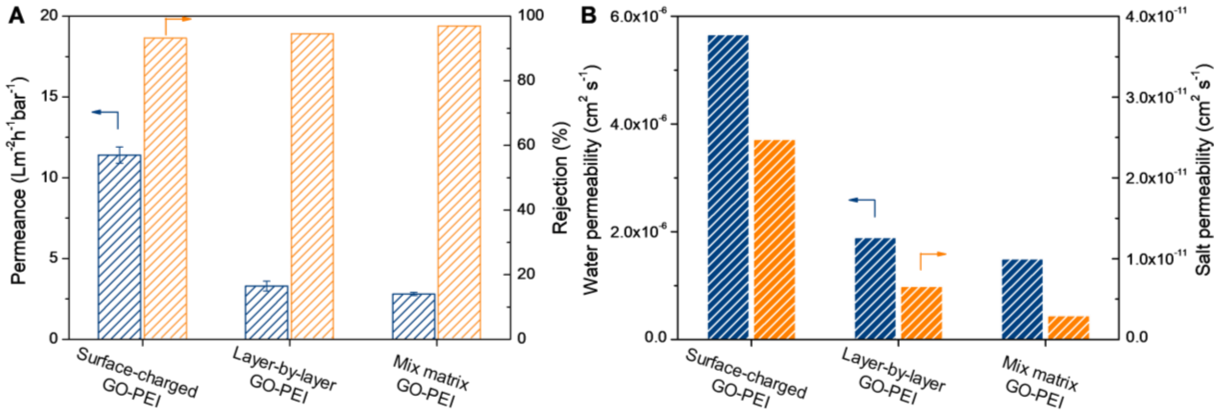

Supplementary Figure 17 (A) Water permeance and $MgCl_2$ rejections, and **(B)** water permeability and salt permeability of surface-charged, layer-by-layer, mixed matrix GO-PEI membranes. Error bars represent standard deviations for 3 measurements.

The $MgCl_2$ rejections of surface-charged, layer-by-layer, mixed matrix GO-PEI membranes keep as high as ~95% and the salt permeabilities of them are lower than 3×10^{-11} cm^2 s^{-1}, because the introduction of PEI providing positively charged membranes surface. However, the water permeance of surface-charged GO-PEI membrane is significantly higher than two other membranes with almost double the water permeability of that of layer-by-layer, mixed matrix GO-PEI membranes, which indicates the superiority of our surface charge controlling strategy. The low water permeance of other membrane configurations is due to no fully utilizing of channel-facilitated water transport within GO laminate.

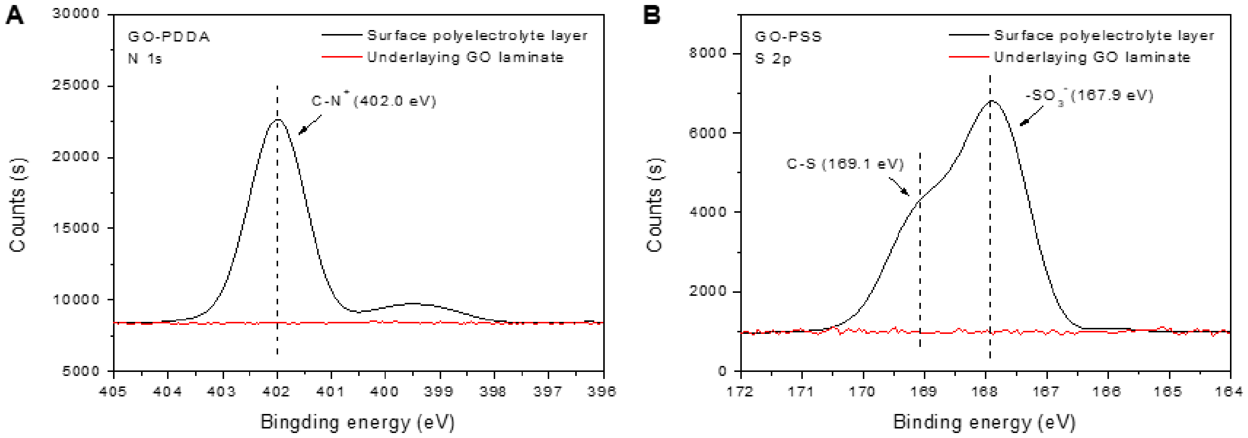

Supplementary Figure 18 (A) XPS N1s spectrum of surface polyelectrolyte layer and underlaying GO laminate in GO-PDDA membrane and **(B)** XPS S2p spectrum of surface polyelectrolyte layer and underlaying GO laminate in GO-PSS membrane.

As confirmed by the XPS spectra of surface polyelectrolyte layer and underlaying GO laminate in surface-charged GO membranes (Supplementary Figure 18) that the characteristic peaks of N and S elements derived from the polyelectrolytes are detected on the surface of the membranes, and are absent when etching into the underlaying GO laminate. These results indicate that the polyelectrolytes only attach on top of the membrane but not enter into the GO laminates.

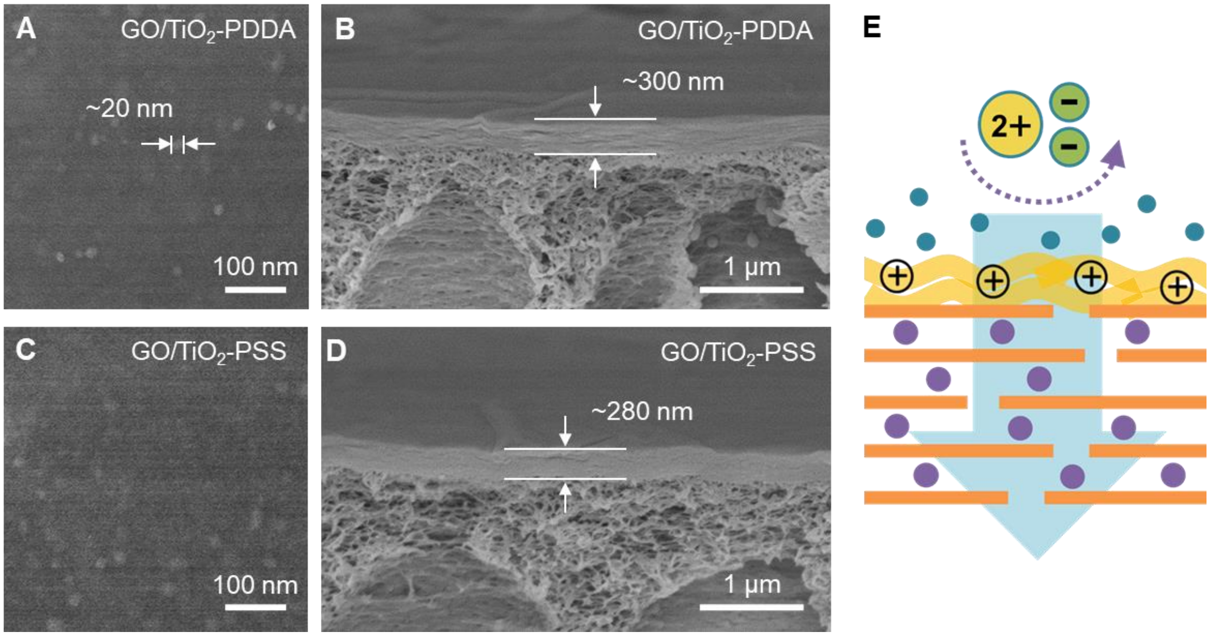

Supplementary Figure 19 (A-D) SEM surface and cross-sectional images of GO/TiO_2-PDDA and GO/TiO_2-PSS membranes (GO deposition amount of 0.5 mg and TiO_2 nanoparticles intercalation amount of 20 wt% with 0.1 wt% polyelectrolyte surface coating). **(E)** Schematic showing the microstructure and separation process of nanoparticles intercalated surface-charged GO membrane.

The membranes morphologies illustrate that the GO laminate with ~20 nm TiO_2 spheres intercalated well-assembles showing ordered laminar structure. The polyelectrolyte layer covers and adheres on the membrane surface. The intercalated nanoparticles can expand and hold the nanochannels within the GO laminate, so that the water permeation through the membrane can be significantly facilitated[11,12].

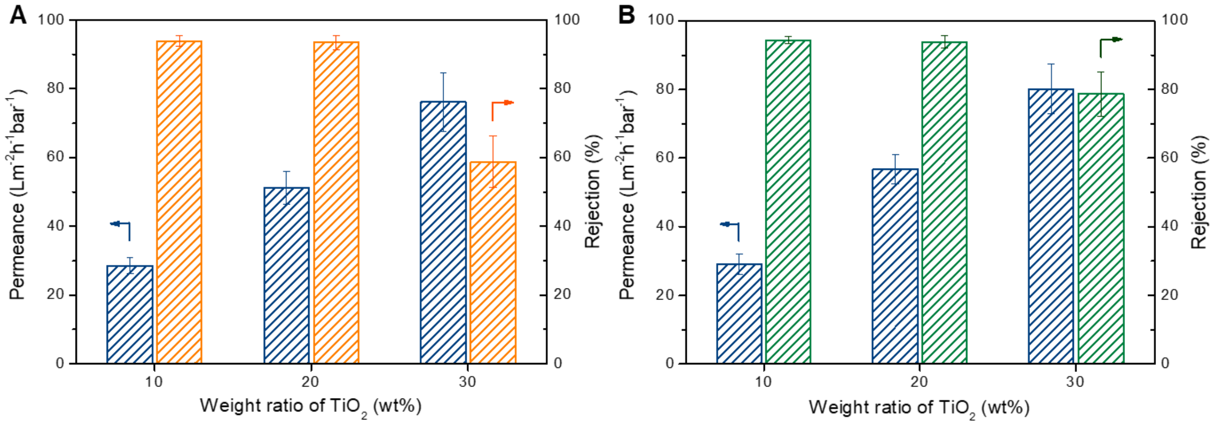

Supplementary Figure 20 (A) Water permeance and $MgCl_2$ rejections of GO/TiO_2-PDDA membranes (GO deposition amount of 0.5 mg and TiO_2 nanoparticles intercalation amount of 10, 20, and 30 wt% with 0.1 wt% PDDA surface coating). **(B)** Water permeance and Na_2SO_4 rejections of GO/TiO_2-PSS membranes (GO deposition amount of 0.5 mg and TiO_2 nanoparticles intercalation amount of 10, 20, and 30 wt% with 0.1 wt% PSS surface coating). Error bars represent standard deviations for 3 measurements.

As observed in Supplementary Figure 20, the water permeance of surface-charged GO/TiO_2 membranes geometrically grows as a function of TiO_2 nanoparticles intercalation amount, confirming the nanoparticle-expanded water channels within GO membranes. However, too much nanoparticles involve disorder and defects of GO assembly, leading to the decay of salt rejections. Therefore, by carefully regulate the nanoparticles intercalation ratio, the surface-charged GO/TiO_2 membranes exhibit nearly 5 times higher water permeance than that of surface-charged GO membranes, as well as similar high salt rejections (GO/TiO_2-PDDA membrane: water permeance of 51.2 Lm^{-2}h^{-1}bar^{-1} with 93.2% $MgCl_2$ rejection; GO/TiO_2-PSS membrane: water permeance of 56.8 Lm^{-2}h^{-1}bar^{-1} with 93.9% Na_2SO_4 rejection).

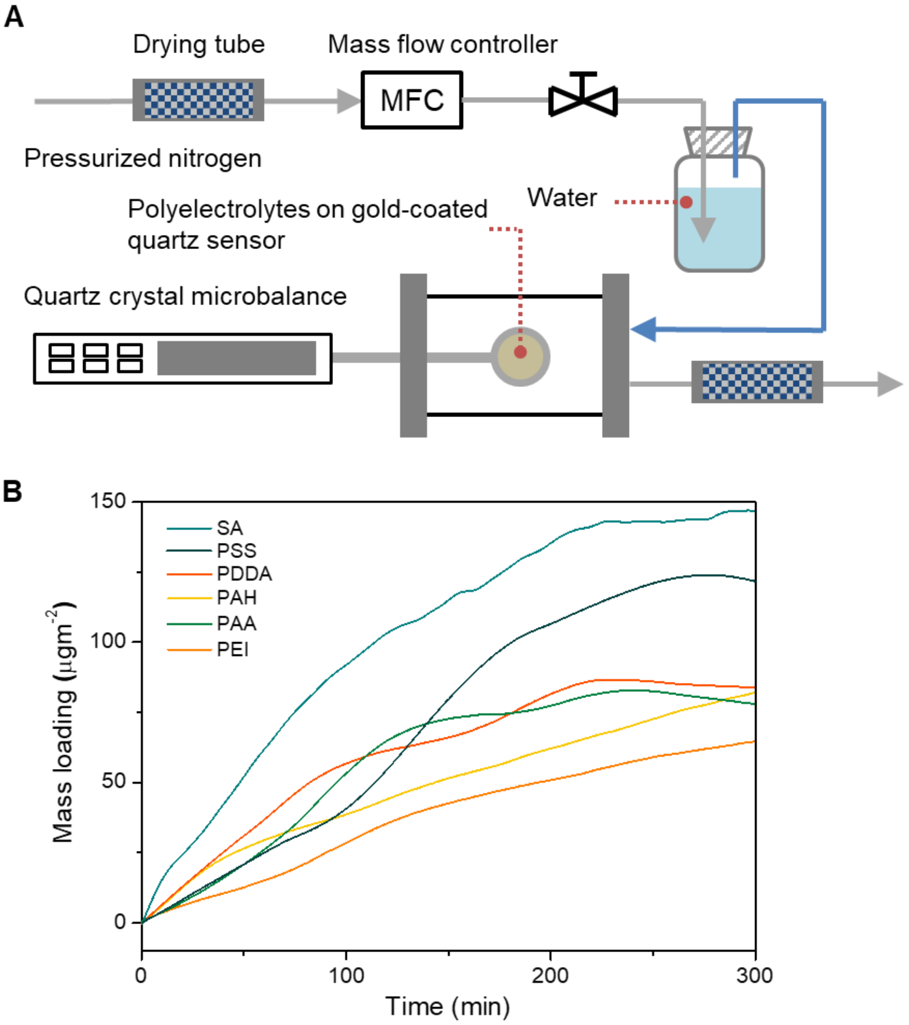

Supplementary Figure 21 (A) Schematic and **(B)** dynamic weight curves of QCM for polyelectrolytes.

The water sorption ability of polyelectrolytes is evaluated by QCM measurement. Air with certain humidity driven into the QCM chamber results in the weight increase of polyelectrolyte layer coated on the quartz sensor. The higher final mass loading indicates better water-sorption ability of polyelectrolyte, leading to the increased membrane hydrophilicity with enhanced water permeance[13].

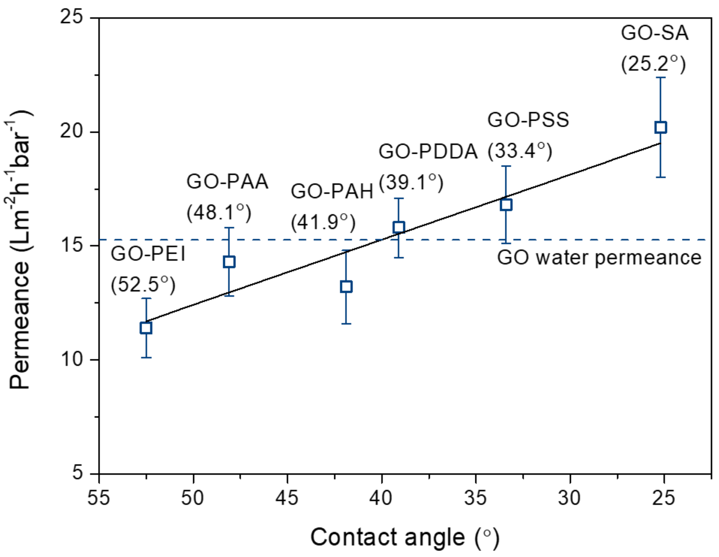

Supplementary Figure 22 Water permeance of surface-charged GO membranes at a function of membrane water contact angles (52.5° of GO-PEI, 48.1° of GO-PAA, 41.9°of GO-PAH, 39.1° of GO-PDDA, 33.4° of GO-PSS and 25.2° of GO-SA membranes). Solid blcak line: best linear fit for water permeance. Dotted blue line: water permeance of pristine GO membrane. Error bars represent standard deviations for 3 measurements.

Because of the abundant hydrophilic groups presented on polyelectrolyte, there is a good linear correlativity between water permeance and water contact angles of surface-charged GO membranes. Especially for GO-SA membrane, the much smaller water contact angle of 25.2° indicates the highly hydrophilicity and strong water-sorption ability of the membrane[13], which is well-agreed the results of QCM measurements.

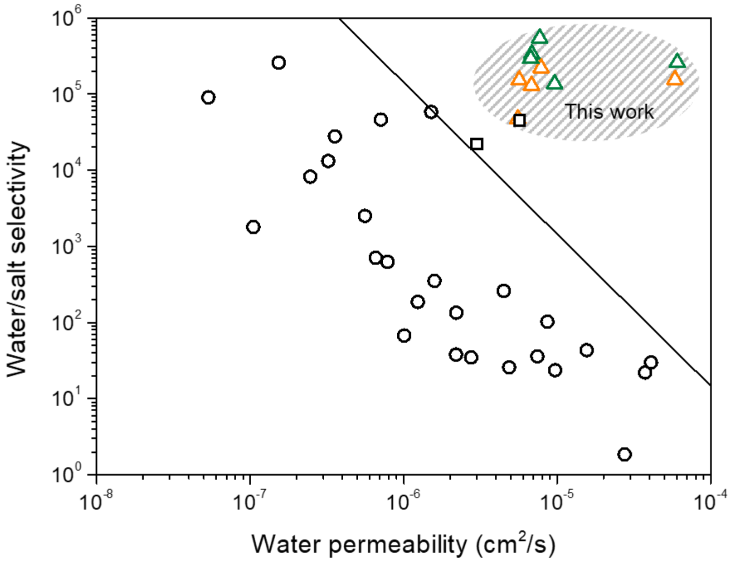

Supplementary Figure 23 Water permeability and water/salt selectivity of surface-charged GO membranes (GO-PDDA, GO-PEI, GO-PAH, TiO_2 intercalated GO-PDDA membranes and GO-SA, GO-PAA, GO-PSS, TiO_2 intercalated GO-PSS membranes) in this work (marked as triangles), as well as comparison with other nanofiltration membranes. These data were obtained from literatures including polymer membranes[14] (marked as circles) and Turing-type PA membranes[15] (marked as squires). Solid line is the permeability-selectivity trade-off for polymeric nanofiltration membranes.

We evaluated water and salts transport properties of our surface-charged membranes and plotted to compare with other nanofiltration membranes. As shown in Supplementary Figure 23, our surface-charged membranes exhibit both high water permeability and water/salt selectivity, surpassing the trade-off of traditional polymeric nanofiltration membranes.

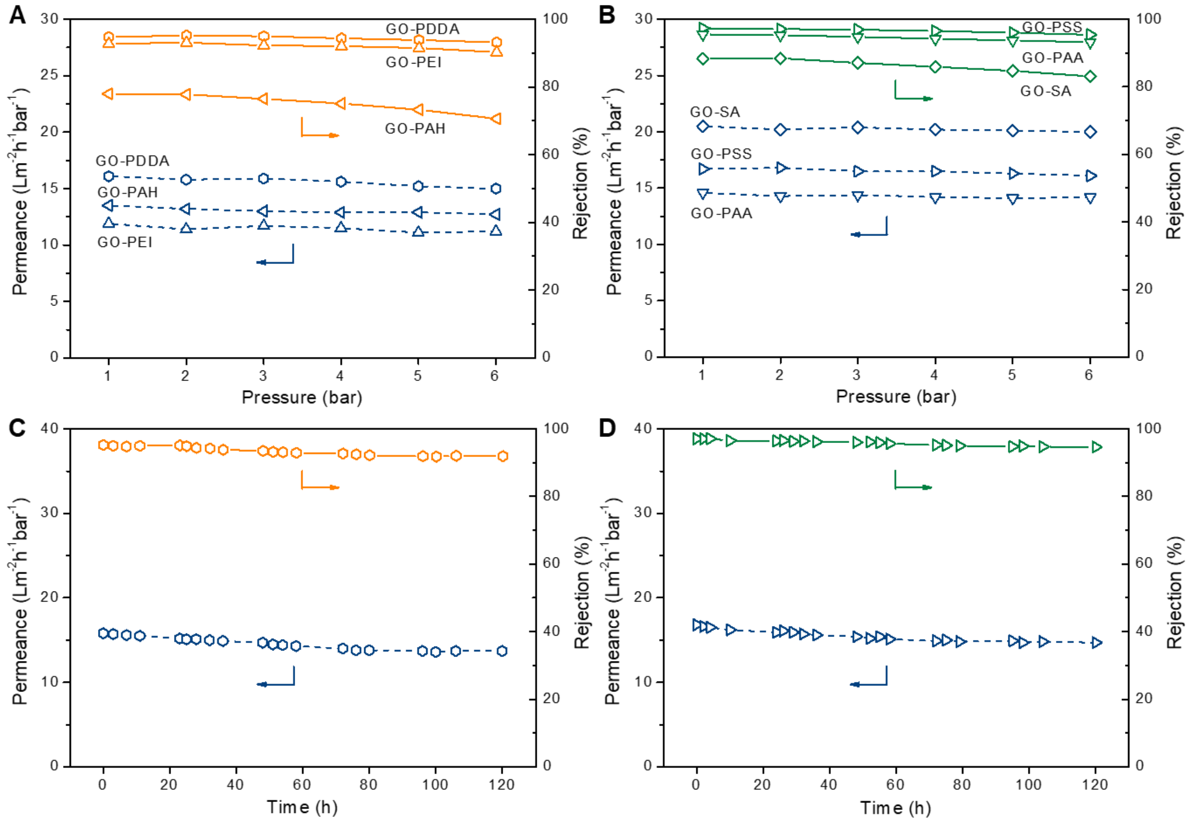

Supplementary Figure 24 (A) Water permeance and $MgCl_2$ rejection of positively charged GO membranes, and **(B)** water permeance and Na_2SO_4 rejection of negatively charged GO membranes under operation pressures ranging from 1-6 bar. **(C-D)** Long-term performance measurements of GO-PAA membrane **(C)** for $MgCl_2$ retention, and GO-PSS membrane **(D)** for Na_2SO_4 retention, over long period of 120 h.

The operation stability is key criterion required for GO-based membranes for practical applications. Thus, we evaluated membrane stability under high-pressure and long-term operations. As confirmed in Supplementary Figure 24, the separation performance of surface-charged GO membranes kept almost stable, indicating that our surface-charged GO membranes can be well-operated over high pressure (⩾6 bar) and long period (⩾120 h).

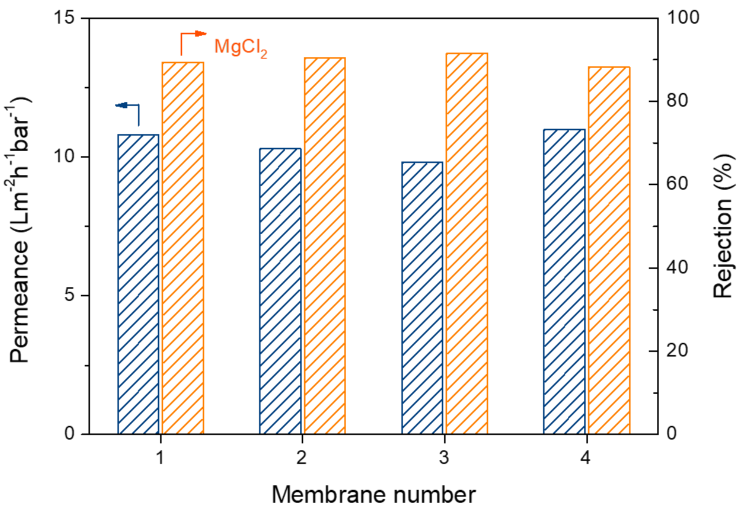

Supplementary Figure 25 Water permeance and $MgCl_2$ rejections of four pieces of membranes incised from different locations at single large-area GO-PDDA membrane with a diameter of 15 cm.

To demonstrate the scalability of our surface charge controlling strategy, a 15 cm-diameter surface-charged GO membrane (effective area of 176.7 cm^2) was fabricated. The homogeneity of the large-area membrane is confirmed by testing the performance of four small pieces of membranes (with a diameter of 4.7 cm) incised from different locations of the large membrane. Water permeance and $MgCl_2$ rejections of each individual membrane are displayed in Supplementary Figure 25, showing similar high separation performance (water permeance of ~10.5 $Lm^{-2}h^{-1}bar^{-1}$ and $MgCl_2$ rejection of ~90%), which is excellent evidence for the scalability of surface charge controlling strategy and the homogeneity of the large-area membrane[16].

Supplementary Table 2 $MgCl_2$ rejection comparisons of positively charged GO membranes in this work with other nanofiltration membranes in literature.

Membrane	Method	Thickness (nm)	Feed concentration	Pressure (bar)	Permeance (LMH/bar)	Rejection (%)
Based-refluxing reduced GO/PVDF[17]	Vacuum filtration	53	20 mM	1	3.26	20
G-CNTm(2:1)/PVDF[18]	Vacuum filtration	40	10 mM	5	11.33	9.6
GO&EDA_HPEI 60K/PDA-PC[19]	Pressurized filtration	69.41	1000 ppm	1	5	96
GO/PSf[20]	Pressurized filtration	150	2000 ppm	15	11	12.5
GO/PVDF[21]	Vacuum filtration	50	3 mM	4	2.4	19.1
TMPyP/GO/PC[22]	Vacuum filtration	-	2000 ppm	8	11.6	45
PEI/GO/h-PAN[23]	Layer-by-layer	59.1	1000 ppm	5	4.2	93.9
GO/PAH/h-PAN[9]	Layer-by-layer	37	6.7 mM	6.9	2	92
Silica/polypiperazine-amide/PES[24]	Interfacial polymerization	42	2000 ppm	6	7.8	50.7
mMSN/PA[25]	Interfacial polymerization	100	5 mM	6	5.4	10
SMWCNT TFN[26]	Interfacial polymerization	100	1000 ppm	6	13.2	62
PD/ZIF-8 templated PA NF[27]	Interfacial polymerization	75	1000 ppm	4	53.5	38
Commercial NF (e.g. DK, DL)	-	-	2000 ppm	6-15	5-14	40-98
DK	-	-	50 ppm	2	7.3	56.5
DL			50 ppm	2	6.2	60.7
GO-PDDA (this work)	Pressurized filtration	130	50 ppm	2	15.8	95.2
GO-PEI (this work)		130			11.4	93.1
GO-PAH (this work)		110			13.2	77.8
GO-PDDA (long term, this work)		130			13.7	91.9
GO/TiO_2-PDDA (this work)		300			51.2	93.2

Supplementary Table 3 Na_2SO_4 rejection comparisons of negatively charged GO membranes in this work with other nanofiltration membranes in literature.

Membrane	Method	Thickness (nm)	Feed concentration	Pressure (bar)	Permeance (LMH/bar)	Rejection (%)
TMC cross-linked GO/PSF[28]	Layer-by-layer	14	10 mM	3.4	8-27.6	26-46
Based-refluxing reduced GO/PVDF[17]	Vacuum filtration	53	20 mM	1	3.26	60
G-CNTm(2:1)/PVDF[18]	Vacuum filtration	40	10 mM	5	11.33	81
GO&EDA_HPEI 60K/PDA-PC[19]	Pressurized filtration	69.4	1000 ppm	1	5	38
GO/PSf[20]	Pressurized filtration	150	2000 ppm	15	11	65
GO/Cellulose[29]	Vacuum filtration	200	10 mM	-	8	67
GO/PVDF[21]	Vacuum filtration	50	3 mM	4	2.4	79.5
GO@PAN[30]	Vacuum filtration	128	-	1	1.8	56.7
TMPyP/GO/PC[22]	Vacuum filtration	-	2000 ppm	8	11.6	88
PEI/GO/h-PAN[23]	Layer-by-layer	59.1	1000 ppm	5	4.2	28
PEI/GO/h-PAN[31]	EF assisted layer-by-layer	77	500 ppm	4	16.4	86.76
GO/ceramic membrane (TiO_2)[32]	Vacuum filtration	10	10 mM	3	58.6	33.7
GO/PAH/h-PAN[9]	Layer-by-layer	37	6.7 mM	6.9	2	68
Silica/polypiperazine-amide/PES[24]	Interfacial polymerization	42	2000 ppm	6	7.8	97.3
mMSN/PA[25]	Interfacial polymerization	100	5 mM	6	5.4	80
SMWCNT TFN[26]	Interfacial polymerization	100	1000 ppm	6	13.2	96.8
MWCNT-OH TFN[33]	Interfacial polymerization	77	2000 ppm	6	6.9	97.6
MWCNT-COOH TF[33]		84			6.2	96.6
MWCNT-NH TFN[33]		71			5.3	96.8
PD/ZIF-8 templated PA NF[27]	Interfacial polymerization	75	1000 ppm	4	53.5	95
Commercial NF (e.g. DK, DL)	-	-	2000 ppm	6-15	5-14	40-98
DK	-	-	50 ppm	2	7.3	93.2

DL			50 ppm	2	6.2	95.8
GO-SA (this work)	Pressure-assisted filtration	125	50 ppm	2	20.2	88.4
GO-PAA (this work)		125			14.3	95.3
GO-PSS (this work)		120			16.8	97.1
GO-PSS (long term, this work)		120			14.7	94.6
GO/TiO_2-PSS (this work)		280			56.8	93.9

Supplementary Table 4 Effective membrane area comparisons of large-area surface-charged GO membrane in this work with other GO-based membranes in literature.

Membrane	Effective membrane area (cm^2)
Based-refluxing reduced GO/PVDF[17]	2.83
G-CNTm(2:1)/PVDF[18]	2.27
GO&EDA_HPEI 60K/PDA-PC[19]	3.14
GO/Cellulose[29]	17.35
GO/PVDF[21]	14.5
GO@PAN[30]	22.9
TMPyP/GO/PC[22]	17.35
PEI/GO/h-PAN[23]	12.5
Slow-deposited GO/AAO[34]	10.5
rGO/MCE[35]	17.35
GO-PDDA (large area, this work)	176.7

Supplementary References

1. A. Paneri, S. Moghaddam, *Carbon* **86**, 245-255 (2015)
2. J. Zhao et al., *J. Mater. Chem. A* **3**, 19980-19988 (2015).
3. L. Nie et al., *Carbohydr. Polym.* **117**, 616-623 (2015).
4. Q. Yang et al., *Nat. Mater.* **16**, 1198 (2017).
5. J. Shen et al., *ACS Nano* **10**, 3398-3409 (2016).
6. W. Tong et al., *Carbon* **94**, 590-598 (2015).
7. A. Rani et al., *Appl. Surf. Sci.* **257**, 4982-4989 (2011).
8. Y. H. Cho et al., *J. Membr. Sci.* **544**, 425-435 (2017).
9. Oh Y. et al., *J. Membr. Sci.* **541**, 235-243 (2017).
10. S. Zheng et al., *ACS Nano* **11**, 6440-6450 (2017).
11. M. Zhang et al., *AIChE J.* **63**, 5054-5063 (2017).
12. K. Guan et al., *J. Membr. Sci.* **542**, 41-51 (2017).
13. K. Huang et al., *Adv. Funct. Mater.* 25, 5809-5815(2015).
14. H. B. Park et al., *Science* **356**, eaab0530 (2017).
15. Z. Tan et al., *Science* **360**, 518-521 (2018).
16. A. Akbari et al., *Nat. Commun.* **7**, 10891 (2016).
17. Y. Han et al., *Adv. Funct. Mater.* **23**, 3693-3700 (2013).
18. Y. Han et al., *ACS Appl. Mater. Interfaces* **7**, 8147-8155 (2015).
19. Y. Zhang et al., *Environ. Sci. & Technol.* **49**, 10235-10242 (2015).
20. Y. Wei et al., *Carbon* **108**, 568-575 (2016).
21. Y. Mo et al., *Desalination* **399**, 40-46 (2016).
22. X. Xu et al., *ACS Appl. Mater. Interfaces* **8**, 12588-12593 (2016).
23. Q. Nan et al., *Appl. Surf. Sci.* **387**, 521-528 (2016).
24. D. Hu et al., *Desalination* **301**, 75-81 (2012).
25. H. Wu et al., *J. Membr. Sci.* **428**, 341-348 (2013).
26. J. Zheng et al., *J. Membr. Sci.* **524**, 344-353 (2017).
27. Z. Wang et al., *Nat. Commun.* **9**, 2004 (2018).
28. M. Hu, B. Mi, *Environmental Science & Technology* **47**, 3715-3723 (2013).
29. G. Liu et al., *Carbon* **110**, 56-61 (2016).
30. J. Wang et al., *ACS Appl. Mater. Interfaces* **8**, 6211-6218 (2016).

31. T. Wang et al., *J. Membr. Sci.* **515**, 125-133 (2016).
32. K. H. Chu et al., *ACS Appl. Mater. Interfaces* **9**, 40369-40377 (2017).
33. S. Xue et al., *ACS Appl. Mater. Interfaces* **8**, 19135-19144 (2016).
34. W. Xu et al., *Nano Lett.* **17**, 2928-2933 (2017).
35. E. Yang et al. *J. Membr. Sci.* **547**, 73-79 (2018).
